# Investigating the content and processes of patient-derived quality of care indicators for those affected by multiple long-term conditions (MLTC): A scoping review protocol

**DOI:** 10.1371/journal.pone.0328016

**Published:** 2025-08-01

**Authors:** Sara Tavares, Arad Reisberg, David Belsey, Ania Henley, Laura Downey

**Affiliations:** 1 Imperial College London, The George Institute Global Health, Scale Space, London, England; 2 Patient Experience Research Centre (PERC), Imperial College London, London, England; 3 The George Institute for Global Health, University of New South Wales, Sydney, New South Wales, Australia; University of Leeds Faculty of Medicine and Health, UNITED KINGDOM OF GREAT BRITAIN AND NORTHERN IRELAND

## Abstract

**Background:**

People living with multiple long-term conditions (MLTC) are reported to have poorer quality of life, worse health outcomes, and higher health-related expenses compared to those with singular chronic health conditions. Living with MLTC is associated with a higher risk of care that is duplicate or unnecessary. Understanding and monitoring the quality of care (QoC) for those with MLTC is imperative to ensure that individuals complex care needs are met, maximising health and wellbeing and minimising harm and social/economic burden. There is paucity on what QoC means in the context of MLTC, which is likely different than a mere amalgamation of quality indicators of each contributory condition. There is even less understanding on how QoC can be measured in a way that meets the specific care priorities of individuals with MLTC. The aim of this review is to systematically map and analyse evidence on what QoC means for those affected by MLTC and the content and processes of any existing QoC indicators in MLTC, developed with patient or caregivers.

**Methods:**

We will systematically search for evidence following a Levac *et al* methodological scoping review process. All eligible studies published in English from 2000 onwards in the following databases will be included: MEDLINE, EMBASE, CINAHL, APA PsycINFO, Global Health and Health Management Information Consortium. Given expected study heterogeneity, a narrative synthesis is anticipated. Our Community Partner Advisory Group will assist in the identification and analysis of relevant evidence.

**Discussion:**

Current evidence shows variations in concepts and approaches when developing, implementing or validating QoC indicators, not always capturing patients’ preferences nor the complex processes required in MLTC care. Clarifying concepts and synthesising evidence-based knowledge in this area will be the first step to inform the development of a project aiming to develop a set of patient-derived QoC indicators for use across multiple settings in the United Kingdom (UK) and beyond.

**Systematic review registration:**

Available on Open Science Framework on https://osf.io/gjr84.

## Introduction

The coexistence of two or more long-term (chronic) conditions [1], often referred to as multimorbidity, is increasingly prevalent as populations live longer, with rates expected to rise substantially over the next 20 years [2]. While the prevalence of multiple long-term conditions (MLTC) increases with age, the absolute number of people living with MLTC appears higher in those under 65 years old [3]. In the United Kingdom (UK) at least two-thirds of adults over 65 are estimated to live with multiple long-term conditions [3] leading to increased healthcare utilisation and costs and resultant strain on health systems [4].

People with MLTC are more likely to experience functional limitations, poorer quality of life, reduced ability to work and lower life expectancy [5,6]. Traditional healthcare models focus on managing individual diseases and struggle to meet the complex needs of these individuals, leading to inefficient service utilisation and gaps in quality of care [7].

Quality indicators are important measurement tools to understand how systems are performing against evidence-based metrics of success, and if change efforts produce desirable results [8]. Although several quality indicators of health have been developed, they focus on single disease-specific indicators (i.e., diagnosis), health outcomes (i.e., mortality or morbidity) or inputs (i.e., medications prescribed) [9]. Due to MLTC complexity and patient’s unpredictable responses to healthcare processes, these measures are not able to fully capture if people’s health improves as a result of care; creating a paradox, where quality markers seem high based on clinical single-disease data, but patient-reported experiences suggest lower quality of care [10].

Although previous reviews have explored quality of healthcare using patient centred-care lenses [11,12], agreement on the best QoC indicators to evaluate the care provided to MLTC individuals remains an urgent need. The challenge of defining universal QoC indicators for these individuals is compounded by the wide range of disease combinations and care requirements [13]. A paradigm shift towards patient-centred care is needed, prioritising pragmatic, multi-layered interventions and indicators [9]. Meaningful QoC indicators must be aligned with individuals’ lived experiences, values, goals and priorities [14] through involvement of patients and caregivers as active stakeholders in designing, delivering, and evaluating healthcare services [11].

To our knowledge, only two other reviews have been published in the area of quality of care in MLTC, but their focus differs from ours as they seek to explore conceptual boundaries, literature gaps [10] or existing clinical guidance for those aged above 65 only [15], regardless of the nature and/ or extent of patient involvement. The need to conduct this review was driven by our community partner advisors with lived experiences of MLTC. Together, we identified the need to map and synthesise existing research on the content and processes of existing patient and caregiver derived QoC indicators and what they prioritise as QoC in MLTC care. Additionally, we saw the importance of synthesising how patients, caregivers, and stakeholders are engaged in quality-of-care initiatives in MLTC care. This review will be the first stage of a multi-step project that aims to co-produce a set of patient and caregiver-derived indicators that could be used in healthcare systems to evaluate and improve care in the UK and potentially beyond.

## Methods

This scoping review protocol was registered on the OSF platform (Open Science Framework) and can be accessed at: https://osf.io/gjr84. The enhanced adapted six-step scoping methodological framework by Levac et al [16], based on Arksey and O’Malley [17], will guide this review with the aim to map the extent, range and nature of research studies focusing on developing QoC indicators in MLTC, using patient- engagement strategies. This framework suggests a consultation stage with stakeholders, patients and caregivers, aligning with the co-production nature of this review and subsequent planned research. Quality assessment of individual studies will not be conducted, as this review aims to provide an overview of all research relevant to our research question [17].

### Identifying the research question

The primary research objective of this scoping review is to identify all primary research on patient-derived QoC indicators for those living with MLTC. Initially we had aimed to report on the implementation and/or evaluation of patient-derived MLTC QoC indicators, but through preliminary searches and our knowledge in this subject, we anticipate a dearth in literature to be able to answer this question. We will seek to answer the following review questions:

1. What does quality of care mean for those affected by MLTC that could inform the development of patient and caregiver derived QoC indicators?
2. What is the content of any existing patient-derived MLTC QoC indicators?
3. What processes have been used to develop patient-derived MLTC QoC indicators?

### Identifying the relevant studies

To achieve a balance between sensitivity and specificity within the search strategy, a three-step search strategy was adopted using the Population, Concept, Context (PCC) format to align study selection with research question. Searches were conducted in seven databases based on the recommendations from Bramer et al [18]; CINAHL (EBSCO), EMBASE (Ovid), MEDLINE (Ovid), PsycINFO (Ovid), Global Health (Ovid), HMIC (Health Management Information Consortium) (Ovid). Since our research questions aim to map research methodology used in the development of potential or established QoC indicators, we did not perform additional searches in the grey literature. To minimise risk of reporting bias and identify any additional ongoing trials relevant to our review, clinical trial registers (ClinicTrial.gov, WHO ICTRP and Clinical Trials Information System) have been searched without any relevant results identified. The reference lists of all included studies will be screened to identify additional publications of relevance and authors of eligible studies will be contacted for any additional information on poorly reported items.

To avoid an overly specific search, both “multimorbidity” and “comorbidity” terms have been considered, despite conceptual differences [5]. We will follow the National Institute for Health and Care Excellence (NICE) definition of multimorbidity, which considers the presence of two or more long-term conditions, including physical or mental health conditions, learning disabilities, symptom complexes, sensory impairments, or alcohol or substance misuse [19]. Likewise, the concept of “quality of care” lacks homogeneity, and its meaning varies between context, geographical area, institutions and individuals [20]. Although we will adopt the definition of a quality indicator as “a measurable item which allows assessment of care” [21], terms such as “indicators”, “measures” and “metrics” will be included in the search strategy, secondary to their interchangeable use in the literature, despite divergent meanings [10,22].

We anticipate identifying a dearth of evidence pertaining to patient-derived QoC indicators for those with MLTC, based on our preliminary scoping searches and broad knowledge of the field. This challenge is complicated by the absence of a universal definition for community or patient engagement and involvement in research. The terminology remains ambiguous, with varying concepts commonly employed, such as “consultation”, “participation”, “collaboration” and “empowerment”. Terms linked to patient-centred care were also sensitive to our search, highlighting an area that has gained substantial attention in evaluating or improving patient involvement within health care system [12,23]. The search strategy tested across the selected databases, devised with the research team, community partners and an experienced research librarian is available in the S1 File. As of March 2025, we have completed the databases searches, and we are in the record screening stages. Data extraction and synthesis are projected to be complete by July 2025. Scoping review results will be published in a peer-reviewed journal, and we will re-run the searches in September 2025, if necessary, as this marks 12 months since the searches were initially conducted.

### Study selection

Searches will be restricted to articles published in English since January 2000, as patient-public active involvement in healthcare planning and quality of care design have emerged in the last two decades [24]. Looking at multimorbidity’s scientific interest, although this has started in the late 90s, it has gained increased focus in the past ten years [25].

We will include primary research studies regardless of their study design alone, provided there is a clear involvement of patients or caregivers to develop QoC indicators or to elicit their priorities with MTLC care. As our research question seeks to explore research methodologies used in this area, editorials, opinion articles, books, correspondences, commentaries and conference abstracts will be excluded. Table 1 provides further insight on the inclusion and exclusion criteria.

**Table 1:** PCC framework inclusion and exclusion criteria.

	Inclusion	Exclusion
**Population**	• Adults (18 years old or over) with diagnosed or self-reported two or more long-term conditions	• Paediatric population or adolescents • Studies describing QoC indicators in populations with only one long-term condition or during acute stages of a disease
**Concept**	• Studies aiming to identify or develop QoC indicators or validate existing QoC with patients and caregivers • Studies that focused on consultation, involvement or co-design QoC indicators with patients and/or caregivers • Studies that elicit patient and/ or caregivers’ priorities with general MLTC care or specific components of MLTC care (i.e., coordination of care)	• Studies developing QoC indicators without explicitly addressing the level or type of patient engagement nor the outcome of patient or caregiver involvement to the delivery, design or evaluation of QoC • Studies focusing on single-disease quality metrics, indicators or improvement strategies • Studies testing or validating instruments to measure specific outcomes of quality, emphasising methodological rigour rather than capturing patients or caregivers’ insights on quality of care
**Context**	• No country-related exclusion • Primary care (outpatient, community settings)	• Not published in English • Acute settings of care looking at in-patient QoC indicators

All citations will be imported into the bibliographic manager Covidence [26] for screening of titles, abstracts, full texts and data extraction. Duplicate citations will be removed automatically and manually whenever necessary. The first author (ST) will assess the eligibility of all studies using a two-step screening process; review of the title/ abstract, followed by a full-text examination of those that appear relevant. Community partners and last author will perform an independent blinded screening of the results for consistency. Both authors (ST, LD) will conduct blinded full-text review of all studies identified for inclusion. Any disagreements will be discussed until a consensus is reached, adjudicated by a third reviewer when necessary. For credibility and reproducibility of findings, the study selection process will be reported according to the Preferred Reporting Items for Systematic Reviews Extension for Scoping Reviews (PRISMA‐ScR) flowchart [27].

### Data Collection and charting the results

All data extraction will be done by the first author, but to ensure consistency between researchers, blinded data extraction for the first five studies will also be done by the last author. An electronic data charting form using Covidence will be created to capture relevant information from each included study. This draft data charting form will be piloted and modified as needed following extraction of the first five reports included in the review. Modifications made to this form will be detailed in the final review manuscript publication. Table 2 includes a broad range of data we consider significant to answer our review questions.

**Table 2:** Data charting form.

Study Characteristics	
Author and Publication year	
Country	
Study Design	
Population Characteristics and recruitment strategy methods	
Healthcare Setting	
Study Aims/ Objectives	
Key Outcomes/ Conclusions	
**Study Methodology and Information on QoC indicators**	
Description and Content of QoC	This will be extracted via qualitative findings of the authors interpretations and verbatims of participants
Data collection methods for the development, implementation or evaluation of QoC indicators	Mapping information whenever possible with the five core steps by Dudley et al. [22], such as importance, validity and reliability, feasibility, usability, assessment of related measures, potential transferability to practice by intended users and any plans for implementation and evaluation.
Data Analysis	
**Patient, public, caregiver or stakeholder involvement in study**	
Nature of Patient and Public Involvement (PPI)	Data will be charted based on Carman et al. [28] framework, identifying level of PPI involvement (i.e., consultation, involvement, partnership or shared leadership) and stage of research (i.e., development, implementation or evaluation).
Expert consensus and stakeholder consultations	Data on consensus methods used to synthesise or making sense of expert opinion (i.e., Delphi process, nominal group methods).

### Collating, summarising and reporting results

The findings will be presented through a structured, three-phase approach. First, the results of the database search will be illustrated using a flowchart. Second, a comprehensive summary of the data charting table will be provided, detailing the content, methodologies, and outcomes of QoC indicators. This will be further enriched by an analysis of the extent and nature of methods employed to engage patients and stakeholders. Finally, given that the majority of the data is expected to be qualitative, a thematic analysis will be conducted to identify recurrent themes on the main domains of quality identified by participants. Data will be systematically organised into categories and domains of quality through a process of familiarisation, code generation, and the grouping of similar-meaning concepts. These themes will be discussed collaboratively between researchers and community advisory partners, and subsequently interpreted through a narrative lens. To guide future discussions with stakeholders and policymakers during next stages of this project, and provide deeper contextual insights on the components of QoC indicators, findings will be mapped against the Donabedian framework on quality of care (structure, process, and outcomes) [29] and allocated, whenever possible, the most relevant Institute of Medicine (IOM) domain of healthcare quality (safe, effective, patient-centred, timely, efficient and equitable) [30].

### Consultation and dissemination

The results of this review will be synthesised in partnership with patient and carer study advisory group members and will guide the next steps of a project aimed at developing a set of patient-derived QoC indicators. Our community partners, authors on this protocol, will continue to be actively involved by editing the manuscript, reviewing search terms, aiding identification of any relevant studies, data screening and synthesis of findings.

## Discussion and conclusion

This scoping review will identify and synthesise existing primary research on patient-derived QoC indicators for those living with MLTC. It will provide a comprehensive overview on what QoC means for patients and caregivers, the characteristics of current MLTC QoC indicators, the research methodologies tested to develop these, and underlying gaps in knowledge. The findings will be subject to an extensive patient and stakeholder consensus process; and will be the first step of a project that seeks to develop a set of meaningful patient-caregiver derived QoC indicators that are pragmatic, relevant, applicable and transferable to multiple care settings. In the long term, this knowledge can potentially inform patient-centred healthcare policies in the UK and beyond, providing a feasible way of providing comparative data for health system monitoring, management and policymaking.

## Supporting information

S1 File Search strategy. (DOCX)
