# Supplementary material for: Investigating the content and processes of patient-derived quality of care indicators for those affected by multiple long-term conditions (MLTC): A scoping review protocol
Source: PLoS One. 2025 Aug 1;20(8):e0328016. doi: 10.1371/journal.pone.0328016 (PMC12316198; doi:10.1371/journal.pone.0328016)
Supplement: S1 File — (DOCX) [file pone.0328016.s001.docx]

**Search strategy**

[Embase Results 2](#_Toc192141868)

[MEDLINE Results 4](#_Toc192141869)

[PsychINFO Results 7](#_Toc192141870)

[HMIC Health Management Information Consortium Results (via Ovid) 9](#_Toc192141871)

[Global Health (via Ovid) 11](#_Toc192141872)

[CINHAL Results 13](#_Toc192141873)

# Embase Results

From January 2000 to 27 September 2024

|  | **Query on Embase (via OVID)** | **Results 27 September 2024** |
| --- | --- | --- |
| 1 | "quality of health care"/ or exp quality indicators, health care/ or quality in healthcare.mp. [mp=title, abstract, heading word, drug trade name, original title, device manufacturer, drug manufacturer, device trade name, keyword heading word, floating subheading word, candidate term word] | 4,744,133 |
| 2 | ((indicator* or measure* or standard* or performance or metric*) adj2 (care or quality)).mp. | 262,666 |
| 3 | quality of care indicators.mp. | 848 |
| 4 | ((indicator* or measure* or metric* or standard*) adj1 develop*).mp. | 14,590 |
| 5 | clinical indicator*.mp. | 12,275 |
| 6 | 1 or 2 or 3 or 4 or 5 | 4,892,642 |
| 7 | (comorbid* or multipatholog* or multimorbid*).mp. | 643,384 |
| 8 | (multiple adj1 (disease* or condition* or diagnos* or ill*)).mp. | 25,645 |
| 9 | multiple chronic conditions.mp. or exp multiple chronic conditions/ | 11,507 |
| 10 | pluripatholog*.mp. | 162 |
| 11 | patient public involvement.mp. or decision making/ or patient participation/ or shared decision making/ | 354,987 |
| 12 | (codevelop* or co-develop* or coproduce or co-produce or coproduction or co-production).mp. | 7,873 |
| 13 | chronic disease/ or chronic care.mp. | 239,153 |
| 14 | 7 or 8 or 9 or 10 or 13 | 895,645 |
| 15 | participatory research/ or patient participation/ or exp community participation/ or stakeholder engagement/ or ((consumer or stakeholder* or community) adj2 (activation or activated or oriented or driven or engage* or empower* or framework* or approach* or guidance or guide or implement* or involve* or participat* or partner* or research* or tool or toolkit* or technique*)).mp. | 134,126 |
| 16 | (Patient-centered care or patient focused care or patient centered care or Patient centred care or patient centeredness or patient centredness or client-centered care or patientcentered care approach or individuali$ed care or person centered or patient focused or progressive patient care model or family-centered care or individual-centered or patient centeredness or patient centredness or client-centered care or patient-centered care approach).mp. [mp=title, abstract, heading word, drug trade name, original title, device manufacturer, drug manufacturer, device trade name, keyword heading word, floating subheading word, candidate term word] | 34,166 |
| 17 | Primary Care/ or exp Primary Health Care/ or exp Community Health Services/ or exp Family Practice/ or exp *community health center/ or public health.mp. | 1,061,243 |
| 18 | (Primary care consultation* or primary care facilit* or primary care facilit* based prevention or Community Health Care or Community Care Networks or community service* or community health service* or community clinic* or community health centre* or community health center*).mp. [mp=title, abstract, heading word, drug trade name, original title, device manufacturer, drug manufacturer, device trade name, keyword heading word, floating subheading word, candidate term word] | 25,815 |
| 19 | (family or physician$).af. or practice$.mp. or primary care.af. or exp Primary Health Care/ or primary.mp. or general pract$.af. or gp.tw. or gps.tw. | 7,557,952 |
| 20 | 17 or 18 or 19 | 8,092,437 |
| 21 | 11 or 12 or 15 or 16 | 482,215 |
| 22 | 6 and 14 and 21 | 8,646 |
| 23 | 20 and 22 | 5,814 |
| 24 | limit 23 to (human and English and yr="2000 -Current" and (adult <18 to 64 years> or aged <65+ years>)) | 2,857 |

# MEDLINE Results

From January 2000 to 27 September 2024

|  | **Query on Ovid MEDLINE(R) ALL** | **Results 27 September 2024** |
| --- | --- | --- |
| 1 | "quality of health care"/ or exp quality indicators, health care/ or quality in healthcare.mp. | 169,743 |
| 2 | ((indicator* or measure* or standard* or performance or metric*) adj2 (care or quality)).mp. | 575 |
| 3 | quality of care indicators.mp. | 1,480 |
| 4 | (indicator* adj1 develop*).mp. | 6,191 |
| 5 | clinical indicator*.mp. | 250,422 |
| 6 | 1 or 2 or 3 or 4 or 5 | 339,845 |
| 7 | (comorbid* or multipatholog* or multimorbid*).mp. [mp=title, book title, abstract, original title, name of substance word, subject heading word, floating sub-heading word, keyword heading word, organism supplementary concept word, protocol supplementary concept word, rare disease supplementary concept word, unique identifier, synonyms, population supplementary concept word, anatomy supplementary concept word] | 131,729 |
| 8 | exp multiple chronic conditions/ or exp comorbidity/ | 16,836 |
| 9 | (multiple adj1 (disease* or condition* or diagnos* or ill*)).mp. [mp=title, book title, abstract, original title, name of substance word, subject heading word, floating sub-heading word, keyword heading word, organism supplementary concept word, protocol supplementary concept word, rare disease supplementary concept word, unique identifier, synonyms, population supplementary concept word, anatomy supplementary concept word] | 650,164 |
| 10 | multiple chronic conditions.mp. or exp chronic disease/ or exp multiple chronic conditions/ | 58 |
| 11 | pluripatholog*.mp. | 977,735 |
| 12 | 7 or 8 or 9 or 10 or 11 | 289,090 |
| 13 | participatory research/ or patient participation/ or exp community participation/ or stakeholder engagement/ or ((consumer or user or patient* or stakeholder* or community) adj2 (activation or activated or oriented or driven or engage* or empower* or framework* or approach* or guidance or guide or implement* or involve* or participat* or partner* or research* or tool or toolkit* or technique*)).mp. | 252,435 |
| 14 | patient public involvement.mp. or decision making/ or patient participation/ or shared decision making/ or Delivery of Health Care/ [mp=title, book title, abstract, original title, name of substance word, subject heading word, floating sub-heading word, keyword heading word, organism supplementary concept word, protocol supplementary concept word, rare disease supplementary concept word, unique identifier, synonyms, population supplementary concept word, anatomy supplementary concept word] | 6,514 |
| 15 | (codevelop* or co-develop* or coproduce or co-produce or coproduction or co-production).mp. [mp=title, book title, abstract, original title, name of substance word, subject heading word, floating sub-heading word, keyword heading word, organism supplementary concept word, protocol supplementary concept word, rare disease supplementary concept word, unique identifier, synonyms, population supplementary concept word, anatomy supplementary concept word] | 43,267 |
| 16 | (Patient-centered care or patient focused care or patient centered care or Patient centred care or patient centeredness or patient centredness or client-centered care or patientcentered care approach or individuali$ed care or person centered or patient focused or progressive patient care model or family-centered care or individual-centered or patient centeredness or patient centredness or client-centered care or patient-centered care approach).mp. [mp=title, book title, abstract, original title, name of substance word, subject heading word, floating sub-heading word, keyword heading word, organism supplementary concept word, protocol supplementary concept word, rare disease supplementary concept word, unique identifier, synonyms, population supplementary concept word, anatomy supplementary concept word] | 287,545 |
| 17 | ((patient* or user* or client* or famil* or individual*) adj1 (care* or centered care or centred care or focus* care or centerdness or centredness or individuali$ed care)).mp. [mp=title, book title, abstract, original title, name of substance word, subject heading word, floating sub-heading word, keyword heading word, organism supplementary concept word, protocol supplementary concept word, rare disease supplementary concept word, unique identifier, synonyms, population supplementary concept word, anatomy supplementary concept word] | 788,318 |
| 18 | 13 or 14 or 15 or 16 or 17 | 5,297,533 |
| 19 | (family or physician$).af. or practice$.mp. or primary care.af. or exp Primary Health Care/ or primary.mp. or general pract$.af. or gp.tw. or gps.tw. | 693,732 |
| 20 | exp Primary Health Care/ or primary care.af. or exp Physicians, Family/ or general pract$.af. or family.in. or family pract$.af. or family physician$.mp. | 929,437 |
| 21 | Primary Care/ or exp Community Health Services/ or exp Family Practice/ or exp *community health center/ or public health.mp. | 55,658 |
| 22 | (Primary care consultation* or primary care facilit* or primary care facilit* based prevention or Community Health Care or Community Care Networks or community service* or community health service* or community clinic* or community health centre* or community health center*).mp. [mp=title, book title, abstract, original title, name of substance word, subject heading word, floating sub-heading word, keyword heading word, organism supplementary concept word, protocol supplementary concept word, rare disease supplementary concept word, unique identifier, synonyms, population supplementary concept word, anatomy supplementary concept word] | 1,027,584 |
| 23 | exp General Practice/ or Primary Care/ or exp Primary Health Care/ or exp Community Health Services/ or exp Family Practice/ or exp *community health center/ or public health.mp. | 1,029 |
| 24 | Home Nursing/cl, mt, og, st [Classification, Methods, Organization & Administration, Standards] | 86 |
| 25 | exp Outpatients/cl [Classification] | 11,046 |
| 26 | exp Ambulatory Care/cl, mt, nu, og, st [Classification, Methods, Nursing, Organization & Administration, Standards] | 939,108 |
| 27 | exp General Practice/ or Primary Care/ or exp Community Health Services/ or exp Family Practice/ or exp *community health center/ or public health.mp. | 5,815,909 |
| 28 | 19 or 20 or 21 or 22 or 23 or 24 or 25 or 26 or 27 | 1,842 |
| 29 | 6 and 12 and 18 and 28  limit 29 to (english language and humans and yr="2000 -Current" and ("adult (19 to 44 years)" or "young adult and adult (19-24 and 19-44)" or "middle age (45 to 64 years)" or "middle aged (45 plus years)" or "all aged (65 and over)" or "aged (80 and over)") and english) | 882 |

# PsychINFO Results

From January 2000 to 27 September 2024

|  | **Query on APA PsycInfo** | | **Results 27 September 2024** |
| --- | --- | --- | --- |
| 1 | ((indicator* or measure* or standard* or performance or metric*) adj2 (care or quality)).mp. | | 25,878 |
| 2 | quality of care indicators.mp. [mp=title, abstract, heading word, table of contents, key concepts, original title, tests & measures, mesh word] | | 103 |
| 3 | clinical indicator*.mp. | | 686 |
| 4 | (quality indicators or (quality control or Quality monitoring or service quality or "Quality of Health Care")).mp. [mp=title, abstract, heading word, table of contents, key concepts, original title, tests & measures, mesh word] | | 19,030 |
| 5 | exp Health Care Quality/ or Health Care Quality.mp. or (exp Quality Control/ or Quality Control.mp.) or (exp "Quality of Care"/ or "Quality of Care".mp.) or (exp Service Quality/ or Service Quality.mp.) or (exp "Quality of Services"/ or "Quality of Services".mp.) or (exp Health Care Quality/ or Health Care Quality.mp.) or (system performance or (quality control or "Quality of Health Care" or health care quality control or quality assurance or Quality monitoring or service quality or quality indicators)).mp. | | 50,322 |
| 6 | 2 or 3 or 4 or 5 | | 50,955 |
| 7 | (comorbid* or multipatholog* or multimorbid*).mp. | | 98,894 |
| 8 | (multiple adj1 (disease* or condition* or diagnos* or ill*)).mp. | | 2,193 |
| 9 | multiple chronic conditions.mp. or exp multiple chronic conditions/ | | 518 |
| 10 | pluripatholog*.mp. | | 9 |
| 11 | (Multimorbidity or multimorbid* or "Any condition in N73.0 specified as chronic" or multiple disease cause).mp. or exp Multiple Disabilities/ or exp Multiple Therapy/ [mp=title, abstract, heading word, table of contents, key concepts, original title, tests & measures, mesh word] | | 4,281 |
| 12 | 7 or 8 or 9 or 10 or 11 | | 103,448 |
| 13 | participatory research/ or patient participation/ or stakeholder engagement/ or ((consumer or stakeholder* or community) adj2 (activation or activated or oriented or driven or engage* or empower* or framework* or approach* or guidance or guide or implement* or involve* or participat* or partner* or research* or tool or toolkit* or technique*)).mp. | | 54,435 |
| 14 | (Empowerment or community empowerment).mp. or (exp Citizen Participation/ or Citizen Participation.mp.) or (exp Client Participation/ or Client Participation.mp.) or (exp Group Participation/ or Group Participation.mp.) or (exp Participation/ or Participation.mp.) or (exp Patient Participation/ or Patient Participation.mp.) or (exp Community Involvement/ or Community Involvement.mp.) or (exp Involvement/ or Involvement.mp.) | | 281,835 |
| 15 | patient public involvement.mp. or decision making/ or patient participation/ or shared decision making/ [mp=title, abstract, heading word, table of contents, key concepts, original title, tests & measures, mesh word] | | 95,160 |
| 16 | (codevelop* or co-develop* or coproduce or co-produce or coproduction or co-production).mp. | | 2,452 |
| 17 | ((patient* or user* or client* or famil* or individual*) adj1 (care* or centered care or centred care or focus* care or centerdness or centredness or individuali$ed care)).mp. | | 55,193 |
| 18 | exp Patient Centered Care/ |  | 991 |
| 19 | (Patient-centered Care or patient focused care or patient centered care or Patient centred care or patient centeredness or patient centredness or client-centered care or patientcentered care approach or individualized care or person centered or patient focused or progressive patient care model or family-centered care or individual-centered or patient centeredness or patient centredness or client-centered care or patient-centered care approach).mp. | | 15,949 |
| 20 | 13 or 14 or 15 or 16 or 17 or 18 or 19 | | 455,424 |
| 21 | 6 and 12 and 20 | | 258 |
| 22 | (family or physician$).af. or practice$.mp. or primary care.af. or exp Primary Health Care/ or primary.mp. or general pract$.af. or gp.tw. or gps.tw. | | 1,880,594 |
| 23 | (Primary care consultation* or primary care facilit* or primary care facilit* based prevention or Community Health Care or Community Care Networks or community service* or community health service* or community clinic* or community health centre* or community health center*).mp. [mp=title, abstract, heading word, table of contents, key concepts, original title, tests & measures, mesh word] | | 28,926 |
| 24 | exp Community Services/ or exp Community Health/ or exp Family Medicine/ or exp Family Physicians/ or exp Primary Health Care/ | | 88,528 |
| 25 | 22 or 23 or 24 | | 1,910,099 |
| 26 | 21 and 25 | | 219 |
| 27 | limit 26 to (human and english language and ("300 adulthood " or 320 young adulthood or 340 thirties or 360 middle age or "380 aged " or "390 very old ") and yr="2000 -Current") | | 149 |

# HMIC Health Management Information Consortium Results (via Ovid)

From January 2000 to 27 September 2024

|  | **Query on HMIC Health Management Information Consortium** | **Results 27 September 2024** |
| --- | --- | --- |
| 1 | exp chronic disease/ or exp Chronic illness/ or exp Morbidity/ or exp multiple disabilities/ | 7,723 |
| 2 | (comorbid* or multipatholog* or multimorbid*).mp. [mp=title, other title, abstract, heading words] | 1,296 |
| 3 | ((multiple adj1 disease*) or condition* or diagnos* or ill*).mp. [mp=title, other title, abstract, heading words] | 55,284 |
| 4 | (multiple chronic condition* or multiple long term condition* or multiple long-term condition*).mp. [mp=title, other title, abstract, heading words] | 98 |
| 5 | 1 or 2 or 3 or 4 | 59,278 |
| 6 | exp Quality assurance in health services/ or exp Quality management/ or exp Quality/ or exp Quality control/ or exp Continuous quality improvement/ or exp Quality standards/ or exp "Quality of patient care"/ or exp Total quality management/ or exp Quality improvement/ or exp "Quality of nursing care"/ or exp Quality assurance/ | 29,590 |
| 7 | exp Performance indicators/ | 2,554 |
| 8 | (((indicator* or measure* or standard* or performance or metric*) adj2 care) or quality).mp. [mp=title, other title, abstract, heading words] | 49,770 |
| 9 | (quality of care indicator* or clinical indicator* or quality in healthcare).mp. [mp=title, other title, abstract, heading words] | 342 |
| 10 | 6 or 7 or 8 or 9 | 54,806 |
| 11 | participatory research/ or exp patient participation/ or exp community participation/ or stakeholder engagement/ or ((consumer or user or patient* or stakeholder* or community) adj2 (activation or activated or oriented or driven or engage* or empower* or framework* or approach* or guidance or guide or implement* or involve* or participat* or partner* or research* or tool or toolkit* or technique*)).mp. | 12,293 |
| 12 | (codevelop* or co-develop* or coproduce or co-produce or coproduction or co-production).mp. [mp=title, other title, abstract, heading words] | 281 |
| 13 | (((patient* or user* or client* or famil* or individual*) adj1 centered care) or centred care or focus* care or centerdness or centredness or individuali$ed care).mp. [mp=title, other title, abstract, heading words] | 2,450 |
| 14 | exp patient centred care/ | 1,559 |
| 15 | exp Collaboration/ or exp Decision making/ | 12,873 |
| 16 | exp patient & public involvement/ or patient public involvement.mp. | 737 |
| 17 | 11 or 12 or 13 or 14 or 15 or 16 | 25,511 |
| 18 | 5 and 10 and 17 | 943 |
| 19 | (Primary care consultation* or primary care facilit* or primary care facilit* based prevention or Community Health Care or Community Care Networks or community service* or community health service* or community clinic* or community health centre* or community health center*).mp. [mp=title, other title, abstract, heading words] | 6,166 |
| 20 | (family or physician$).af. or practice$.mp. or primary care.af. or primary.mp. or general pract$.af. or gp.tw. or gps.tw. | 110,874 |
| 21 | exp Nursing homes/ or exp General practice/ or exp community services/ or home care/ or exp Out patients/ or exp Ambulatory care/ or exp Public health/ or exp community health services/ or exp primary care/ or exp Community health care/ or exp primary care/ | 49,479 |
| 22 | 19 or 20 or 21 | 133,556 |
| 23 | 18 and 22 | 561 |
| 24 | limit 23 to (yr="2000 -Current" and english) | 470 |

# Global Health (via Ovid)

From January 2000 to 27 September 2024

|  | **Query on Global Health** | **Results 27 September 2024** |
| --- | --- | --- |
| 1 | multimorbidity.mp. or chronic diseases.sh. or morbidity.sh. or comorbidity.sh. | 82,628 |
| 2 | multiple long term conditions.mp. or multiple chronic condition*.mp. | 298 |
| 3 | multiple disease*.mp. | 877 |
| 4 | (multiple diagnos* or multiple chronic ill* or morbidity pattern* or pluripatholog*).mp. | 586 |
| 5 | multimorbid*.mp. | 1,780 |
| 6 | comorbid*.mp. or comorbidity.sh. | 43,365 |
| 7 | 1 or 2 or 3 or 4 or 5 or 6 | 108,548 |
| 8 | ((indicator* or measure* or standard* or performance or metric*) adj2 (care or quality)).mp. | 28,723 |
| 9 | quality of care indicators.mp. | 119 |
| 10 | (indicator* adj1 develop*).mp. | 615 |
| 11 | healthcare quality.mp. or "quality of care".sh. | 12,100 |
| 12 | clinical indicator.mp. | 188 |
| 13 | quality standard*.mp. or quality standards.sh. | 6,788 |
| 14 | quality treatment* or quality measure*).mp. | 1,737 |
| 15 | system performance.mp. and health services.sh. and health care.sh. | 408 |
| 16 | 8 or 9 or 10 or 11 or 12 or 13 or 14 or 15 | 40,574 |
| 17 | participatory research/ or patient participation/ or exp community participation/ or stakeholder engagement/ or ((consumer or user or patient* or stakeholder* or community) adj2 (activation or activated or oriented or driven or engage* or empower* or framework* or approach* or guidance or guide or implement* or involve* or participat* or partner* or research* or tool or toolkit* or technique*)).mp. | 47,253 |
| 18 | (patient public involvement or decision making or patient participation or shared decision making or Empowerment or community empowerment or citizen Participation or Client Participation or Group Participation or Patient Participation or Community Involvement).mp. [mp=abstract, title, original title, broad terms, heading words, cabicodes words] | 47,871 |
| 19 | (codevelop* or co-develop* or coproduce or co-product* or coproduct*).mp. [mp=abstract, title, original title, broad terms, heading words, cabicodes words] | 1,642 |
| 20 | Patient-centered Care or patient focused care or patient centered care or Patient centred care or patient centeredness or patient centredness or client-centered care or patientcentered care approach or individuali$ed care or person centered or patient focused or progressive patient care model or family-centered care or individual-centered or patient centeredness or patient centredness or client-centered care or patient-centered care approach).mp. [mp=abstract, title, original title, broad terms, heading words, cabicodes words] | 1,733 |
| 21 | (((patient* or user* or client* or famil* or individual*) adj1 centered care) or centred care or focus* care or centerdness or centredness or individuali$ed care).mp. [mp=abstract, title, original title, broad terms, heading words, cabicodes words] | 1,418 |
| 22 | (social participation or stakeholders or community involvement or communities or participation).sh. | 51,501 |
| 23 | (empowerment and patients and health care).sh. | 114 |
| 24 | patient cent*.mp. | 3,056 |
| 25 | 17, 18 or 19 or 20 or 21 or 22 or 23 or 24 | 121,723 |
| 26 | (Primary care consultation* or primary care facilit* or primary care facilit* based prevention or Community Health Care or Community Care Networks or community service* or community health service* or community clinic* or community health centre* or community health center*).mp. [mp=abstract, title, original title, broad terms, heading words, cabicodes words] | 12,350 |
| 27 | (general Practic* or Primary Care or community Health Servic* or family Practic* or public health or amulatory care or outpatient* or nursing home* or family care or family physician* or family practice* or community practice* or primary care or primary health care or community care).mp. [mp=abstract, title, original title, broad terms, heading words, cabicodes words] | 500,469 |
| 28 | (health services or primary health care or general practitioners).sh. | 139,082 |
| 29 | exp long term care/ or outpatient services/ or exp community care/ | 6,600 |
| 30 | 26 or 27 or 28 or 29 or 30 or 31 | 574,700 |
| 31 | 7 and 15 and 25 and 30  limit 31 to (english language and yr="2000 -Current") | 155 |

# CINHAL Results

From January 2000 to 27 September 2024

|  | **Query on CINHAL (Ebsco Host)** | **Results 27 September 2024** |
| --- | --- | --- |
| S1 | comorbid* OR multimorib* OR or multiple chronic condition* OR comorbid* disorder* OR chronic condition* or long term condition* or chronic disease* OR multiple chronic disease* OR multiple chronic ill* OR multiple health problem* OR multiple disease* or multiple condition* |  |
| S2 | (MH "Chronic Disease+") OR "multiple chronic diseases or multiple chronic conditions |  |
| S3 | multiple N1 disease* or condition* or diagnos* or ill* |  |
| S4 | S1 OR S2 OR S3 |  |
| S5 | indicator* OR measure* OR standard* OR performance OR metric* N1 Care OR quality |  |
| S6 | (MH "Clinical Indicators") |  |
| S7 | (MH "Quality of Health Care+") OR "healthcare quality" |  |
| S8 | indicator* N1 develop* |  |
| S9 | S5 OR S6 OR S7 OR S8 |  |
| S10 | (MM "Stakeholder Participation") OR "Patient Participation+") OR (MH "Family Involvement (Iowa NIC)") OR "participation or engagement or involvement" |  |
| S11 | MH "Patient Participation+") OR " (patient participation or patient involvement or patient empowerment or patient engagement) OR patient partnership OR participatory action research OR community engagement" |  |
| S12 | "( community participation or community engagement or community involvement ) OR ( community participation and empowerment ) OR ( community participation in the planning and management )" |  |
| S13 | (MH "Stakeholder Participation") OR "patient public involvement" |  |
| S14 | (MH "Patient Centered Care") OR (MH "Family Centered Care+") OR "(patient-centered care or client centered care or person-centered care ) OR ( Patient centred care or patient centeredness or patient centredness or client-centered care or patientcentered care approach or individualized care or person centered or patient focused or progressive patient care model or family-centered care or individualcentered or patient centeredness or patient centredness |  |
| S15 | S10 OR S11 OR S12 OR S13 OR S14 |  |
| S16 | primary health care or public health care or community care or primary care or primary care nursing or family medicine or family practice or general practice |  |
| S17 | (MH "Ambulatory Care") OR (MH "Ambulatory Care Facilities+") OR (MH "Outpatient Service") OR (MH "Outpatients") OR (MH "Home Health Care+") OR (MH "Community Health Centers+") OR (MH "Hospitals, Community") OR (MH "Home Visits") OR (MH "Home Nursing") OR (MH "Nurse Specialist Service (Saba CCC)") OR (MH "Clinical Nurse Specialists+") OR (MH "Multidisciplinary Care Team+") OR (MH "Patient Care Plans+") OR (MH "Disease Management+") OR (MH "Health Care Delivery+") OR (MH "Community Health Services) |  |
| S18 | TI ( ("ambulatory clinic" or "outpatient clinic*" or "virtual clinic*" or ((remote or home) n2 monitor*) or "home care" or "home visit*" or "house call*" or "specialist nurse*" or "clinic*" or "multi-disciplinary team*" or "multidisciplinary team*" or "outreach" or "disease management program*" or "community led care" or "community service*" or "community health service*" or "community clinic*" or "community health centre*" or "community health center*" or " pharmacy led care" or "pharmacy services” |  |
| S19 | S16 AND S17 AND S18 |  |
| S19 | S4 AND S9 AND S15 AND S19  Limiters - Publication Date: 20000101-; English Language; Human; Age Groups: Adult: 19-44 years, Middle Aged: 45-64 years, Aged: 65+ years, Aged, 80 and over, All Adult | 1824 |
