# Supplementary material for: Investigating the content and processes of patient-derived quality of care indicators for those affected by multiple long-term conditions (MLTC): A scoping review protocol
Source: PLoS One. 2025 Aug 1;20(8):e0328016. doi: 10.1371/journal.pone.0328016 (PMC12316198; doi:10.1371/journal.pone.0328016)
Supplement: Peer review history

# Editor report: Decision Letter 0

**DOI:** 10.1371/journal.pone.0328016.r001

**Authors:** Olga Lainidi

Investigating the content and processes of patient-derived quality of care indicators for those affected by multiple long-term conditions (MLTC): A scoping review protocol

PONE-D-25-12073

Dear Dr. Tavares,

We’re pleased to inform you that your manuscript has been judged scientifically suitable for publication and will be formally accepted for publication once it meets all outstanding technical requirements.

Within one week, you’ll receive an e-mail detailing the required amendments. When these have been addressed, you’ll receive a formal acceptance letter and your manuscript will be scheduled for publication.

An invoice will be generated when your article is formally accepted. Please note, if your institution has a publishing partnership with PLOS and your article meets the relevant criteria, all or part of your publication costs will be covered. Please make sure your user information is up-to-date by logging into Editorial Manager at Editorial Manager®  and clicking the ‘Update My Information' link at the top of the page. If you have any questions relating to publication charges, please contact our Author Billing department directly at authorbilling@plos.org.

If your institution or institutions have a press office, please notify them about your upcoming paper to help maximize its impact. If they’ll be preparing press materials, please inform our press team as soon as possible -- no later than 48 hours after receiving the formal acceptance. Your manuscript will remain under strict press embargo until 2 pm Eastern Time on the date of publication. For more information, please contact onepress@plos.org.

Kind regards,

Olga Lainidi

Academic Editor

PLOS ONE

Journal Requirements:

1. Thank you for stating in your Funding Statement:
  “Imperial College NIHR Biomedical Research Centre (BRC) supports the development of this project and this review. ST is supported by the NIHR DCAF doctoral fellowship. The views expressed in this publications are those of the author(s) and not necessarily those of the NIHR or the Department of Health or the Department of Health and Social Care.”
  Please provide an amended statement that declares *all* the funding or sources of support (whether external or internal to your organization) received during this study, as detailed online in our guide for authors at http://journals.plos.org/plosone/s/submit-now. Please also include the statement “There was no additional external funding received for this study.” in your updated Funding Statement.
  Please respond by return e-mail so that we can amend your financial disclosure and competing interests on your behalf.
2. Thank you for stating the following financial disclosure:
  “Imperial College NIHR Biomedical Research Centre (BRC) supports the development of this project and this review. ST is supported by the NIHR DCAF doctoral fellowship. The views expressed in this publications are those of the author(s) and not necessarily those of the NIHR or the Department of Health or the Department of Health and Social Care.”
  Please state what role the funders took in the study. If the funders had no role, please state: "The funders had no role in study design, data collection and analysis, decision to publish, or preparation of the manuscript."
  If this statement is not correct you must amend it as needed.
  Please respond by return e-mail so that we can amend your financial disclosure and competing interests on your behalf.

# Editor report: Acceptance letter

**DOI:** 10.1371/journal.pone.0328016.r002

**Authors:** Olga Lainidi

PONE-D-25-12073

PLOS ONE

Dear Dr. Tavares,

I'm pleased to inform you that your manuscript has been deemed suitable for publication in PLOS ONE. Congratulations! Your manuscript is now being handed over to our production team.

At this stage, our production department will prepare your paper for publication. This includes ensuring the following:

* All references, tables, and figures are properly cited

* All relevant supporting information is included in the manuscript submission,

* There are no issues that prevent the paper from being properly typeset

You will receive further instructions from the production team, including instructions on how to review your proof when it is ready. Please keep in mind that we are working through a large volume of accepted articles, so please give us a few days to review your paper and let you know the next and final steps.

Lastly, if your institution or institutions have a press office, please let them know about your upcoming paper now to help maximize its impact. If they'll be preparing press materials, please inform our press team within the next 48 hours. Your manuscript will remain under strict press embargo until 2 pm Eastern Time on the date of publication. For more information, please contact onepress@plos.org.

If we can help with anything else, please email us at customercare@plos.org.

Thank you for submitting your work to PLOS ONE and supporting open access.

Kind regards,

PLOS ONE Editorial Office Staff

on behalf of

Dr. Olga Lainidi

Academic Editor

PLOS ONE
